# Chimeric Music Reveals an Interaction of Pitch and Time in Electrophysiological Signatures of Music Encoding

**DOI:** 10.1523/JNEUROSCI.2083-24.2025

**Published:** 2025-12-19

**Authors:** Tong Shan, Edmund C. Lalor, Ross K. Maddox

**Affiliations:** ^1^Department of Biomedical Engineering, University of Rochester, Rochester, New York 14627; ^2^Department of Neuroscience, University of Rochester, Rochester, New York 14627; ^3^Del Monte Institute for Neuroscience, University of Rochester, Rochester, New York 14627; ^4^Department of Otolaryngology-Head & Neck Surgery, Kresge Hearing Research Institute, University of Michigan, Ann Arbor, Michigan 48109

**Keywords:** beat perception, expectation encoding, music perception, neural tracking, temporal response function

## Abstract

Pitch and time are the essential dimensions defining musical melody. Recent electrophysiological studies have explored the neural encoding of musical pitch and time by leveraging probabilistic models of their sequences, but few have studied how the features might interact. This study examines these interactions by introducing “chimeric music,” which pairs two distinct melodies and exchanges their pitch contours and note onset times to create two new melodies, distorting musical pattern while maintaining the marginal statistics of the original pieces’ pitch and temporal sequences. Through this manipulation, we aimed to dissect the music processing and the interaction between pitch and time. Employing the temporal response function framework, we analyzed the neural encoding of melodic expectation and musical downbeats in participants with varying levels of musical training. Our findings from 27 participants of either sex revealed differences in the encoding of melodic expectation between original and chimeric stimuli in both dimensions, with a significant impact of musical experience. This suggests that decoupling the pitch and temporal structure affects expectation processing. In our analysis of downbeat encoding, we found an enhanced neural response when participants heard a note that aligned with the downbeat during music listening. In chimeric music, responses to downbeats were larger when the note was also a downbeat in the original music that provided the pitch sequence, indicating an effect of pitch structure on beat perception. This study advances our understanding of the neural underpinnings of music, emphasizing the significance of pitch–time interaction in the neural encoding of music.

## Significance Statement

Listening to music is a complex and multidimensional auditory experience. Recent studies have investigated the neural encoding of pitch and timing sequences in musical structure, but they have been studied independently. This study addresses the gap in understanding of how the interaction between pitch and time affects their encoding. By introducing “chimeric music,” which decouples these two melodic dimensions, we investigate how this interaction influences the neural activities using EEG. Leveraging the temporal response function framework, we found that structural violations in pitch–time interactions impact musical expectation processing and beat perception. These results advance our knowledge of how the brain processes complex auditory stimuli like music, underscoring the critical role of pitch and time interactions in music perception.

## Introduction

Pitch and time are the basis of musical melody (Lerdahl and Jackendoff, 1996; Tillmann, 2012; Lerdahl, 2019). The interaction between these dimensions can influence the perception of each other (Krumhansl, 2000; Prince et al., 2009; Prince, 2011). For example, the detection of pitch deviants depends on temporal structure regularities (Jones et al., 1982, 2002; Herbst and Obleser, 2019). Rhythm processing is also affected by the tonal structure and the predictability of pitch (Tillmann and Bharucha, 2002; Keitel et al., 2025). A simple example of pitch and rhythm's perceptual interaction is that a completely isochronous melody in a triple time signature could still be heard as a waltz because of a tendency to return to specific notes on the first beat of a three-beat measure. This interplay exemplifies the complexity of music as a multidimensional auditory experience.

When processing music, expectancy emerges from listeners’ experience with contextually relevant musical structure, influencing the processing of subsequent events (Huron, 2008; Tillmann, 2012). Neuroimaging studies have shown that violating expectations triggers neural responses to unexpected musical patterns.

Recently, temporal response functions (TRF) have been used to study the neural tracking of continuous stimuli by modeling ongoing brain activity based on specific stimulus features (Ding and Simon, 2012; Crosse et al., 2016). TRF studies have provided insights on the neural tracking of melodic expectations (where “melody” encompasses both pitch and timing) in continuous music (Di Liberto et al., 2020a; Marion et al., 2021; Kern et al., 2022; Bianco et al., 2024). These investigations reveal that the melodic expectation process is inherently active while humans listen to music, with quantified features like musical note surprise and uncertainty being key components. These components are computed from probabilistic models like the Information Dynamics of Music (IDyOM; Pearce, 2005; Pearce and Wiggins, 2012), which computes expectation for both pitch and time. Recent work using IDyOM demonstrated that TRFs reflect expectancies only in timing in nonhuman primates (Bianco et al., 2024) but in both pitch and timing in humans (Di Liberto et al., 2020a). However, because IDyOM models pitch and timing probabilities separately without modeling their interaction and because pitch and timing expectancies are inextricably coupled in the natural stimuli, it is still not clear how the human brain separately processes expectations based on pitch and timing or how they interact.

Another aspect of music processing involves meter perception, reflecting perceived temporal regularity within music (Snyder and Krumhansl, 2001; Grahn, 2012) at multiple scales. This temporal structure perception also establishes an expectancy that the events occurring on strong beats (e.g., downbeats that starts a measure) are processed as more salient than those “off the beat” (Geiser et al., 2010; Schaefer et al., 2011; Tierney and Kraus, 2013, 2015; Bouwer et al., 2014; Fitzroy and Sanders, 2015; Moon et al., 2020). However, few studies have explored how pitch's structure influences this temporal structure perception, and those that exist rely on repetitive, simple music excerpts rather than continuous melodies (Lelo-de-Larrea-Mancera et al., 2017; Zhang et al., 2019).

**Figure 1.**
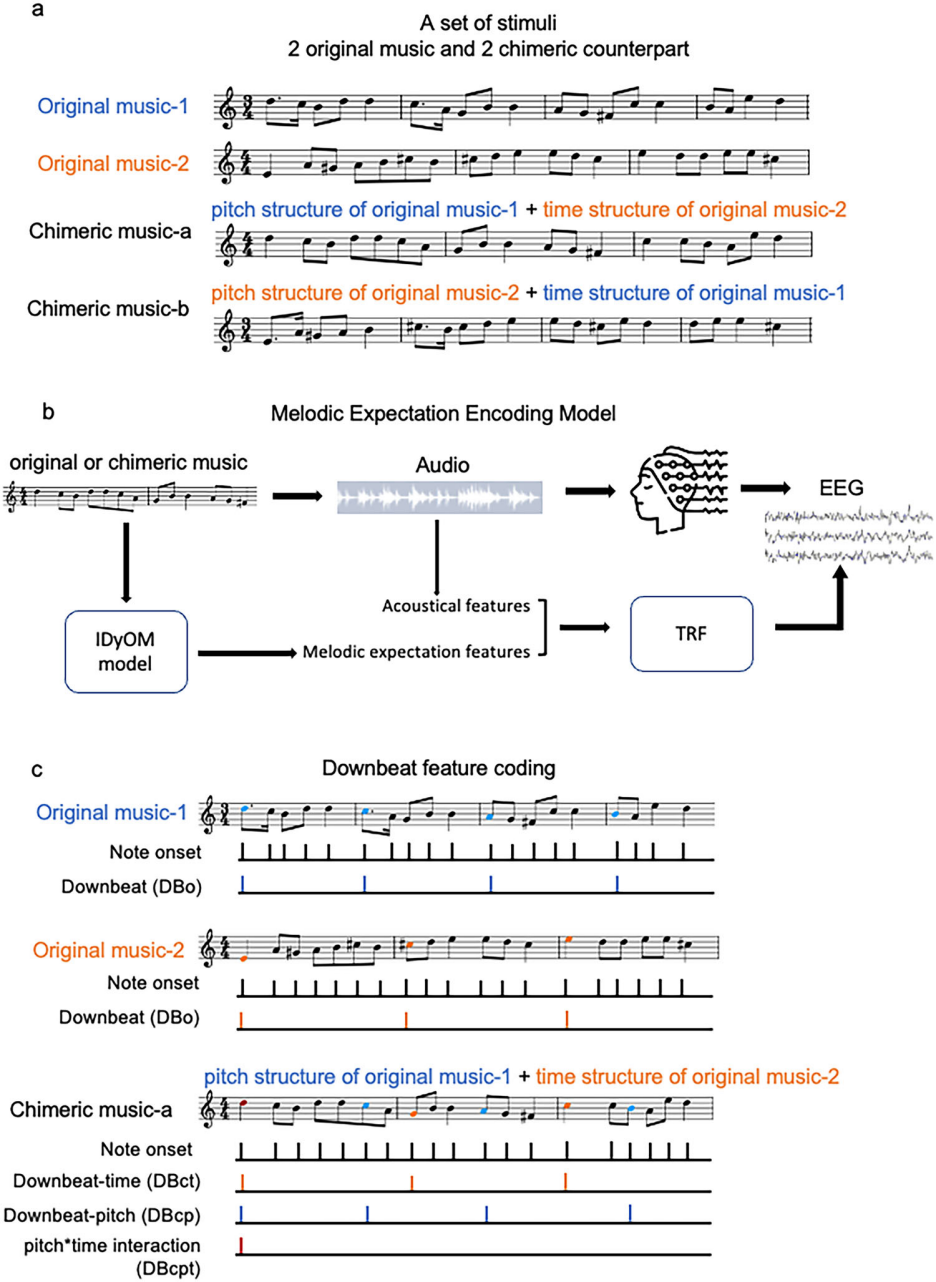
Overview of the research framework. ***a***, An example set of stimuli consists of two original music and their two chimeric music counterparts. The chimeric music-a has the pitch structure of original music-1 and time (rhythm) structure of original music-2. The chimeric music-b has the pitch structure of original music-2 and time structure of original music-1. ***b***, TRF paradigm for the melodic expectation encoding. Participants listen to the monophonic music, including original and chimeric music. Acoustical features including spectral flux and note onsets were computed from the music. The IDyOM model was used to calculate the note-level melodic expectation features for pitch and time, including pitch surprisal, pitch entropy, time surprisal, and time entropy. The acoustical features (A) and the melodic expectation features (M) were used as the regressors in the TRF model. ***c***, The regressor illustration for the downbeat encoding model. For original music, binary-coded impulse trains served as a regressor that indicates when the note onset and downbeat (DBo) occur. For chimeric music-a, in addition to the note onset, the downbeat-time (DBct) was inherited from the original music-2 downbeat regressor (marked orange), whereas the downbeat-pitch (DBcp) was from the original music-1 downbeat regressor (marked blue). The interaction term (DBcpt) was when the note is both DBct and DBcp (marked red).

Given that the perceptual interaction of pitch and rhythm is clear, we hypothesize that the neural encoding of each should be influenced by the other and visible using EEG as altered TRFs. To better understand this interaction, we introduce a new auditory stimulus: chimeric music (Fig. 1*a*), which exchanges the pitch and rhythm structures from different natural music pieces, resulting in new compositions that sound less structured and predictable. By decoupling the pitch and time dimensions and violating their traditional linkage while preserving the marginal statistics of the original pieces, it provides a way to study pitch–time interaction.

Our initial investigation focused on the neural encoding of melodic expectation within the TRF framework (Fig. 1*b*), following the methodologies of previous studies (Di Liberto et al., 2020a; Kern et al., 2022; Bianco et al., 2024). We compared the neural tracking of the original music and their chimeric counterparts. Moreover, we studied the influence of pitch structure on the time structure through downbeat encoding. The downbeat, defined as the first beat of a measure and typically the strongest metrical accent (Jehan, 2005), serves as a focal point for this analysis. We used a TRF framework alongside a set of binary-coded indicator features according to the note onset and metrical structure (Fig. 1*c*). We found both the neural encoding of melodic expectation and musical downbeats were influenced by pitch–time interactions in musical structure.

## Methods and Materials

### Participants

Twenty-seven people (8 males, 17 females, and 2 others) participated in this experiment with an age of 22.9 ± 3.9 (mean ± STD) years. All participants had audiometric thresholds of 20 dB HL or better from 500 to 8,000 Hz. Participants self-reported having normal or corrected to normal vision. Following the experimental session, participants completed a self-reported musicianship survey (see our OpenNeuro Dataset).

All participants gave informed consent and received compensation for their time. Data collection was conducted under a protocol approved by the University of Rochester Subjects Review Board (#66988).

### Original music and chimeric music stimuli

Monophonic European folksongs from the Essen Folksong Collection (Schaffrath, 1995) corpus were used as the stimuli in this study. We selected pieces that had durations longer than 25 s, excluding those with less discernable melody lines or many repetitions. The selected pieces were then converted to MIDI, a format which encodes the note value, onset, and duration of each note. The specific pieces in this collection are not widely known and are likely not familiar to participants (although we did not collect specific data on this), but they likely carried an air of familiarity for participants who grew up in the Western music tradition.

To explore the interaction between musical pitch and time, we created a new stimulus called “chimeric music.” Note pitch sequence and the note onset times sequence were extracted from each piece using python package *Mido* (Bjørndalen and Doursenaud, 2023). Chimeric music was created by combining the pitch sequence from one song with the note time sequence of another, forming pairs as illustrated in Figure 1*a*. Pairs were made through the following approach: music pieces were first categorized into two beat structure groups based on their time signatures—either triple beats (3/4, 3/8, and 6/8 time signatures) or duple/quadruple beats (2/4, 4/4, and 4/8 time signatures). Pieces were then paired such that each pair consisted of one from each beat group. When fusing the pitch and time, the note matching always started with the beginning, ensuring the same context prior in each domain. To avoid significant duration loss, the paired pieces had close to the same number of notes, so that the synthesized chimeric music had similar duration to the original. This pairing strategy was designed to disrupt the structured melodic patterns by combining pitch contours associated with triple time signatures with duple or quadruple beat timing or vice versa.

Each stimulus set contained two original matched music and two chimeric music counterparts. In total, 33 sets of stimuli were present in this study, including 66 original music and their 66 chimeric music counterparts. Both original and chimeric music stimuli were rendered into WAV files by *FluidSynth* using its default acoustic grand piano SoundFont with a sampling rate of 48 kHz. All music notes in all music maintained uniform velocity (i.e., same intensity), and the overall amplitudes of the stimuli were normalized to a root mean square of 0.01, ensuring consistent loudness across stimuli.

### Stimulus presentation and procedure

During the experimental session, participants were seated in a sound-isolating booth facing a 24 inch BenQ monitor, where they passively listened to the provided stimuli. Each stimulus was presented at a consistent average level of 60 dB SPL. The stimulus audio was delivered through ER-2 insert earphones (Etymotic Research) connected to an RME Babyface Pro digital sound card (RME). The stimulus presentation for the experiment was controlled by a python script using the *expyfun* package (Larson et al., 2014). There were in total 132 music trials with duration ranging from 27 to 50 s, resulting in a total duration of the experiment ∼77 min. The stimuli were played in a random order to participants. Following each music trial, participants were asked to rate their preference on the monitor for the heard piece on a scale of 1 through 7, with 1 indicating strongly disliked and 7 strongly liked.

### EEG data acquisition and preprocessing

Subcortical and cortical EEG signals were simultaneously recorded using the ReCorder software from BrainVision (Brain Products). For the acquisition of subcortical signals, passive Ag/AgCl electrodes were placed frontocentrally (FCz, noninverting), left and right earlobes (A1, A2, inverting references), and the frontal pole (Fpz, ground). These electrodes were connected to two EP-Preamp amplifiers connected to an actiCHamp Plus recording system (both from Brain Products). For the acquisition of cortical signals, a 32-channel configuration was employed using active electrodes, arranged according to the international 10–20 system. The 32-channel system was directly connected to the ActiCHamp plus. To ensure good signal quality, the impedance of all electrodes was verified to be below 10 kΩ before starting the recordings. Both signals were recorded at a high sampling rate of 10 kHz.

Analyses were performed using the *MNE-python* package (Gramfort et al., 2013; Ablin et al., 2018; Larson et al., 2023). The subcortical signal was high-pass filtered offline at 1 Hz using a first-order causal Butterworth filter to remove the slow drift. We also used a second-order infinite impulse response notch filter to remove 60 Hz line noise and its multiples with a width of 5 Hz (Shan et al., 2024). The left and right channels were averaged as the final subcortical EEG signal. The cortical signal was high-passed at 1 Hz and low-passed at 8 Hz with bidirectional zero-phase FIR filter and downsampled to 125 Hz. All channels were rereferenced to the average of the two mastoid channels TP9 and TP10 to highlight the auditory responses (Luck, 2014). To repair the eye movement and blink artifact, we conducted an independent components analysis (ICA), and we excluded epochs with excessive variance from subsequent analysis.

### Cortical EEG analysis

The TRF (Lalor et al., 2009; Ding et al., 2014; Crosse et al., 2016), a time-resolved regression analysis technique, was employed in this study to compute the neural tracking of music features using the mTRF toolbox (Crosse et al., 2016) and its adapted python version (Bialas et al., 2023). The TRF has been widely used to map the cortical neural activity evoked by continuous auditory stimuli, such as speech and music (Ding et al., 2014; Di Liberto et al., 2015, 2020a; Crosse et al., 2016). The TRF was computed by a linear regression to estimate model weights that optimally represent the relationship between neural activity and single or multiple stimulus features. The derived TRF function acts as a filter that is interpretable in sensor space and time lag, offering insights comparable to those provided by event-related potentials (ERPs).

For this study, we aimed to explore different aspects of neural tracking of music by fitting two distinct groups of TRF models. These models were designed to incorporate various groups of features, as detailed in the subsequent sections.

#### Melodic expectation encoding

The first aspect we explored was the melodic expectation encoding in original and chimeric music and their differences. The expectation features were note-level markers derived from the IDyOM model (Pearce, 2005; Pearce and Wiggins, 2012), which is a hidden Markov model that learns the stimulus statistical patterns and predicts the probabilistic structure of symbolic sequences within specific stimulus domains. For a given musical context, IDyOM estimates the likelihood of the next musical note based on the prior n−1 sequence (i.e., *n*-gram context). The model gives the output of the sequences in two aspects: (1) surprisal (*S*) or information content, reflecting the unexpectedness of the current note $x_{t}$ at time t given the preceding notes $x_{t-n}^{t-1}$, calculated as follows:

$$S(x_{t})=-\ln\;P(x_{t}|x_{t-n}^{t-1}),$$

and (2) the entropy (*E*), reflecting the uncertainty of the previous context before the $x_{t}$ happens, computed across all notes in the set of possible notes (x∈K) as follows:

$$E(x_{t})=\underset{x\in K}{\sum }\;P(x|x_{t-n}^{t-1})S(x).$$

These computations were derived from different musical dimensions or viewpoints. In this study, we focused on the pitch (IDyOM viewpoint *cpitch*) and interonset interval (IDyOM viewpoint *ioi*) to analyze the pitch and time dimensions, denoted as Sp (pitch surprisal), Ep (pitch entropy), St (time surprisal), and Et (time entropy).

The IDyOM model can incorporate both a short-term model (STM) and long-term model (LTM), where the STM is trained on the current stimulus trial and the LTM is trained on broader corpus material simulating statistical learning through lifetime musical enculturation. In this study, we used melodic expectation features derived from a combination of STM and LTM (termed “both” model in IDyOM), in line with previous work (Di Liberto et al., 2020a). The LTM was trained on European folksongs also from Essen Folksong Collection (Schaffrath, 1995) corpus excluding the songs that were used as stimuli.

Following the previous studies investigating the melodic expectation encoding in music (Di Liberto et al., 2020a; Kern et al., 2022; Bianco et al., 2024), an acoustical model (denoted as A) was fitted with continuous spectral flux and the note onset (a binary indicator with impulses where a note onset occurred), which served as a baseline model. We then extended this model forming the melodic expectation TRF model (denoted as AM), which incorporated both the aforementioned acoustic features and the expectation features derived from IDyOM (Fig. 1*b*). To explore the contribution of melodic expectation features from the pitch and time dimensions individually, we also tested two reduced models from the full AM model including only pitch dimension features (Sp/Ep) or time dimension features (St/Et), denoted as AMp and AMt, respectively.

Notably, given that the sequence of the pitch and time were consistent across both original and chimeric music (i.e., same context in each dimension), the resultant expectation features Sp/Ep and St/Et were identical. This design allowed TRFs to be fit using the same feature vectors for both stimulus types, with potential TRF differences indicating an effect of the pitch–time decoupling inherent to the chimeric stimuli.

Ridge regression was used to train the TRF models to avoid overfitting (lambda values ranges from 10^−3^ to 10^3^). We used leave-one-out cross–validation (across trials) to evaluate the TRF models on unseen data. The lambda for each TRF model and for each participant providing the best prediction of unseen data was chosen. The average Pearson's correlation between the predicted EEG signal and the recorded real EEG signal across all channels was used as the metric to assess the TRF model's prediction accuracy.

#### Music downbeat encoding

The second aspect we explored was downbeat encoding in original and chimeric music. Initially, we established a note onset regressor, made as an impulse train where each musical note occurrence was marked by a unit impulse. For the original music pieces, a downbeat regressor (DBo) was similarly created as an impulse train, coding the onsets of downbeat notes as 1 and all other timepoints as 0 (the nonzero points in this regressor were thus a subset of the onset regressor). In the case of chimeric music, we delineated two types of downbeats: downbeat-pitch (DBcp) represented notes that were originally downbeats in the piece whose pitch contour was included in the chimeric stimulus, and downbeat-time (DBct) represented notes that were downbeats in the piece whose rhythmic sequence was used. Note that the downbeat-time notes were also the downbeat note by definition for the chimeric stimulus, as downbeats are temporally defined. We also created a pitch × time interaction regressor (DBcpt) as an impulse train indicating the note is simultaneously a downbeat-pitch note and downbeat-time note. An example of these regressors is shown in Figure 1*c*. Chimeric music-a has the pitch contour of original music-1 and rhythm contour of original music-2. The sixth note (C5 highlighted with blue) was marked as downbeat-pitch originating from original music-1, while the eighth note (G4 highlighted with orange) marked as downbeat-time originating from original music-2.

In the downbeat encoding analysis, we were interested in comparing neural encoding through the TRF weights for each of the downbeat regressors. Therefore, we trained TRF models from all the trials for original or chimeric music for each participant. Since the regressors were all impulse trains, we used the ordinary least squares regression instead of ridge regression in order to preserve the interpretability of the TRF weights.

### Statistical analysis

#### Melodic expectation encoding TRF analysis

For the melodic expectation encoding TRF analysis, a linear mixed-effect model was applied using the statsmodels package (Seabold and Perktold, 2010) to analyze the prediction accuracy (measured by the correlation, Pearson's *R*) with the TRF model types (A or AM), the stimulus category (original or chimeric music), the musicianship (participants’ formal music training years), the preference ratings, and their interaction. These factors were considered as fixed effects, while individual participants and the stimulus set were treated as random effects. The model formula used was as follows:

$$\begin{array}{rl} & r\;∼\mathrm{TRF}\;\mathrm{model}+\mathrm{stim}\;\;\mathrm{category}+\mathrm{musicianship}+\mathrm{preference}\\ & +\mathrm{TRF}\;\mathrm{model}\;*\;\mathrm{stim}\;\mathrm{category}+\mathrm{musicianship}\;*\;\mathrm{stim}\;\;\mathrm{category}\\ & +\mathrm{preference}\;*\;\mathrm{stim}\;\mathrm{category}+(1|\mathrm{participant})+(1|\mathrm{stim}\;\mathrm{set}). \end{array}$$

To further assess the enhancement effect of melodic expectation, we calculated the difference in prediction accuracy between each melodic expectation model and the acoustic baseline model within each participant and stimulus set. A subsequent linear mixed-effect model was fitted with TRF model types of contrast (AMp−A, AMt−A, AM−A), the stimulus category (original or chimeric music), the musicianship, the preference ratings, and their interaction terms as fixed effect, again considering participant and the stimulus set as random effects. The formula for this analysis was as follows:

$$\begin{array}{rl} & \Delta r\;∼\mathrm{TRF}\;\mathrm{model}+\mathrm{stim}\;\mathrm{category}+\mathrm{musicianship}+\mathrm{preference}\\ & +\mathrm{TRF}\;\mathrm{model}\;*\;\mathrm{stim}\;\mathrm{category}+\mathrm{musicianship}\;*\;\mathrm{stim}\;\;\mathrm{category}\\ & +\mathrm{preference}\;*\;\mathrm{stim}\;\mathrm{category}+(1|\mathrm{participant})+\left(1|\mathrm{stim}\;\mathrm{set}\right). \end{array}$$

Post hoc paired *t* tests with Holm–Bonferroni correction were performed to determine the statistical differences between each model type and stimulus category.

Additionally, we visualized the derived TRF weights for each regressor, utilizing a permutation test with clustering to identify significant differences in TRF weights between original and chimeric music. The test was performed using a two-sided one–sample permutation *t* test with threshold-free cluster enhancement (TFCE) implemented in MNE-Python, with 1,000 subject-level sign–flipping permutations and a spatiotemporal adjacency matrix linking neighboring sensors and consecutive time points. Family-wise error was controlled by comparing observed TFCE scores against the permutation null distribution, and points with corrected *p* < 0.05 were considered significant, with no minimum cluster size required.

#### Downbeat encoding TRF analysis

To determine downbeat encoding, we displayed the TRF weights for each regressor, employing permutation tests with clustering for statistical inference (the same permutation test as mentioned above). For original music, TRF weights for note onset and DBo regressors were contrasted against zero (the null response). Similarly, for chimeric music, TRF weights for note onset, DBcp, DBct, and DBcpt were also contrasted against zero. A comparison between the note onset TRF weights from original and chimeric music was conducted, as well as the DBo and DBct TRF weights.

Pearson's correlation analysis was also used to explore the relationship between the amplitude of derived TRF weights and the length of formal musical training (in years) of the participants while applying false discovery rate (FDR) correction for multiple comparisons.

### Subcortical EEG analysis

In the derivation of the subcortical auditory brainstem response (ABR), a deconvolution paradigm was used as described in Shan et al. (2024). These responses are also calculated as TRFs, but with some key differences. They were recorded with passive electrodes and special preamps suitable for the ABR (Brain Products EP-Preamp) and analyzed at a much higher sampling rate of 10,000 samples/s. The input features were created by passing the stimuli through a model of the auditory periphery which outputs the auditory nerve firing rate. The resulting TRFs show responses at much shorter latencies that mirror the standard ABR morphology.

The grand-averaged ABR waveforms from original and chimeric music were then compared. Statistical inference was determined by timepoint-wise Wilcoxon signed-rank test with FDR correction.

## Results

### Expectation effect is larger in original than in chimeric music

**Figure 2.**
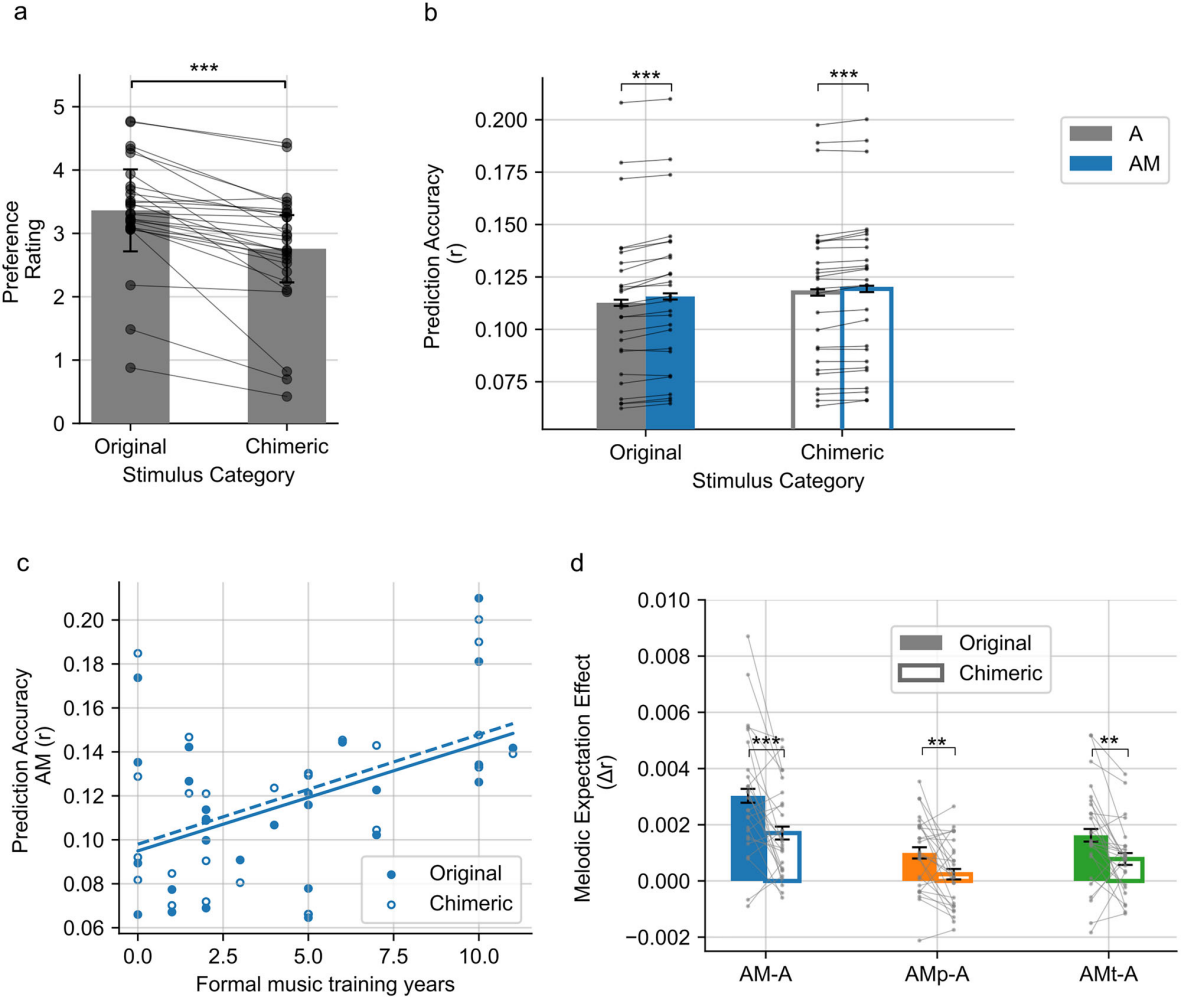
***a***, Preference rating. The bars show the averaged rating of each stimulus category. Error bar indicates ±1 SEM. ***b***, The TRF prediction accuracy of A and AM model. ***c***, The correlation between the TRF prediction accuracy of the AM model and the music expertise as in formal music training years. ***d***, The melodic expectation effect as the prediction accuracy difference (Δ*r*) between each melodic expectation models (AM, AMp, AMt) and the baseline acoustic model (A). Connected data points show the values from each participant. *p < 0.05; **p < 0.01; ***p < 0.001.

Our first analysis confirmed a significant preference for original music over chimeric music (*p* < 0.001, paired *t* test; Fig. 2*a*).

We used the TRF model to explore the neural tracking of melodic expectation during listening to the original and chimeric music. We compared the prediction accuracy of the four TRF models between the two stimulus categories. These models were acoustical feature only model (A) and model combing both acoustics and melodic expectation features from IDyOM's model (AM) as described in the Methods and Materials section. The prediction accuracies for each trial were obtained using leave-one-out cross–validation. A linear mixed-effect model was subsequently fitted to examine the influence of stimulus category (original or chimeric music), model types (A or AM), participants’ musical training, and preference rating on prediction accuracy, as summarized in Table 1.

**Table 1.** The fitted mixed-effect linear model results for prediction accuracy (Pearson's *r*)

	Coeff	*z*	*p*
Intercept (Original Music A)	0.092	35.074	<0.001
Chimeric music	0.011	4.418	<0.001
AM	0.003	2.772	0.006
Chimeric × AM	−0.001	−0.858	0.391
Musicianship	0.005	13.325	<0.001
Chimeric × musicianship	−0.000	−0.616	0.538
Preference	−0.000	−0.224	0.823
Chimeric × preference	−0.002	−3.348	0.001

Original music and Model A are the baselines for the term category and model.

In setting our baseline (intercept) as model A for original music, our analysis revealed significant enhancements in prediction accuracy for the TRF models incorporating both acoustic and melodic expectation features with AM terms tested significant (*p* < 0.001). This improvement in predictive accuracy aligns with previous research (Di Liberto et al., 2020a; Kern et al., 2022), demonstrating that including melodic expectation features better explains neural activity during music listening. Notably, the AM terms were significant for both original and chimeric music, as depicted in Figure 2*b*, suggesting that the expectation process is active even when the normal associations of pitch and temporal structure are violated.

Furthermore, our findings indicated that length of formal musical training is correlated with prediction accuracy in general (*p* < 0.001 for the musicianship term). A subsequent post hoc analysis confirmed that the musicianship is significantly correlated with the prediction accuracy for both original and chimeric music for both A and AM models as shown in Figure 2*c*. Interestingly, while preference alone did not significantly influence prediction accuracy, the interaction between preference and stimulus type exhibited a small but significant negative effect, indicating slightly stronger neural tracking of less preferred chimeric music.

To delve deeper into the melodic expectation effect, we examined the prediction accuracy difference (Δ*r*) between the melodic expectation model (AM) and the baseline acoustic model (A), as well as the contribution of the pitch or time dimension features (AMp-A or AMt-A). The results, summarized in Table 2, show a significant negative coefficient for chimeric music, indicating a greater overall melodic expectation effect for original music compared with chimeric music. Post hoc comparisons further revealed that prediction improvement offered by the melodic expectation features was significant for all AM, Amp, and AMt models (Fig. 2*d*). This suggests that both pitch dimension features and time dimension features contributed to the melodic expectation effect. Additionally, while preference alone did not significantly influence the melodic expectation effect, the interaction between preference and stimulus type exhibited a small but significant positive effect, indicating a slightly stronger melodic expectation effect of preferred chimeric music.

**Table 2.** The fitted mixed-effect linear model results for the expectation effect (Δr)

	Coeff	*z*	*p*
Intercept (Original Music AM-A)	0.003	7.397	<0.001
Chimeric Music	−0.003	−5.028	<0.001
AMp-A	−0.002	−6.691	<0.001
AMt-A	−0.001	−4.897	<0.001
Chimeric × AMp-A	0.000	1.029	0.304
Chimeric × AMt-A	0.001	1.362	0.173
Musicianship	−0.000	−0.450	0.653
Chimeric × musicianship	0.000	1.893	0.058
Preference	−0.000	−0.685	0.493
Chimeric × preference	0.000	3.181	0.001

Original music and AMp-A are the baselines for the term category and model.

When comparing TRF weight waveforms of each regressor from the AM model between original and chimeric music, no significant differences were identified between the two stimulus categories (all *p* > 0.05, permutation test with cluster; refer to Fig. S1).

### Downbeat notes elicit distinct responses

**Figure 3.**
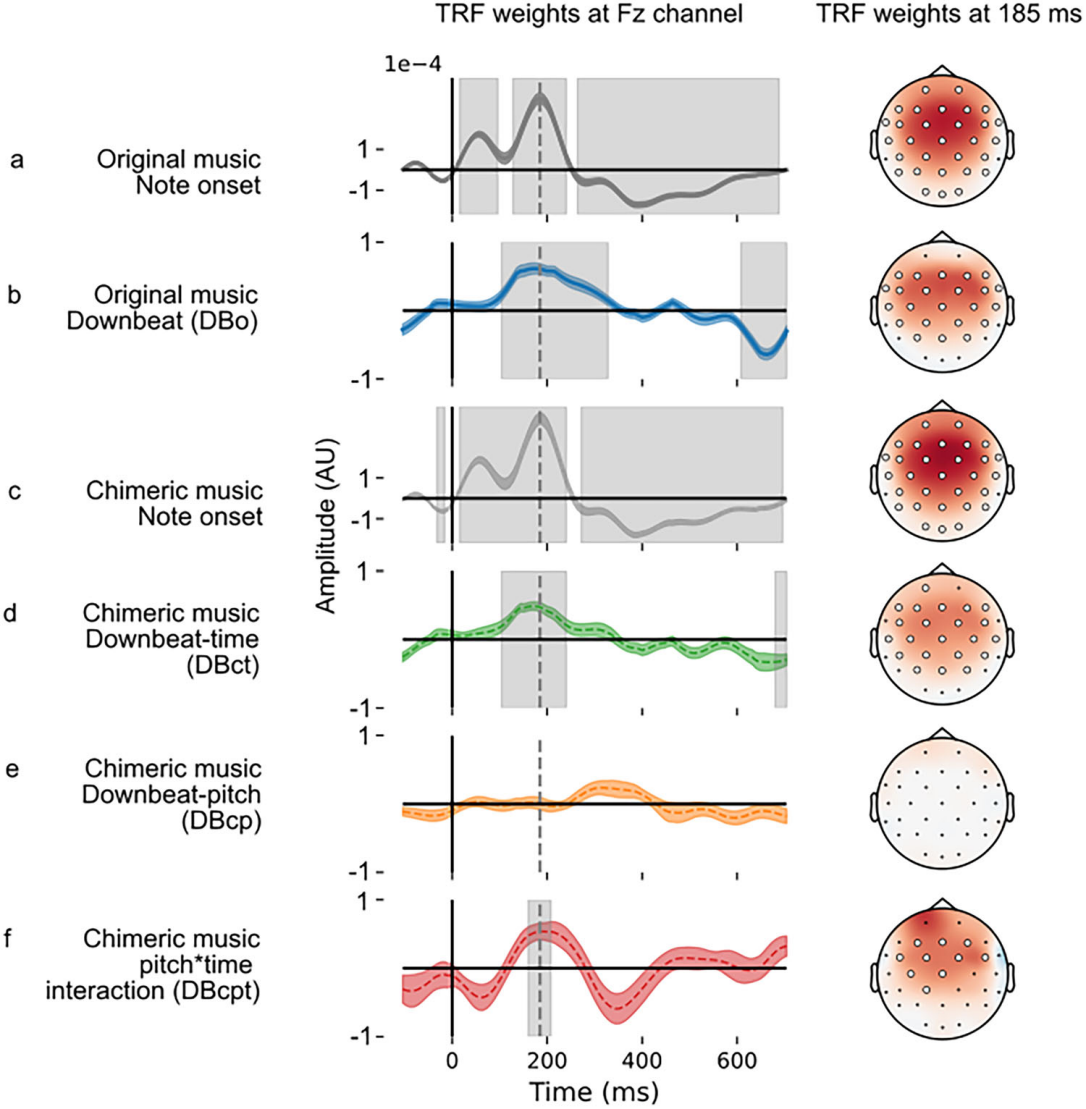
TRF weights in the downbeat encoding model for original music note onset (***a***), DBo (***b***), chimeric music note onset (***c***), DBct (***d***), DBcp (***e***), and DBcpt (***f***). The left side is the TRF weights at Fz channel. Colored-shaded area shows ±1 SEM. The gray area shows the significant time range. The right side is the topography of the TRF weighs at the peak time point, 185 ms. The larger circles show the significant channels.

We next explored whether the neural encoding of downbeats (note onsets that occur on the first beat of the measure), defined in time domain, is influenced by another music domain, pitch, leading to another aspect of the music processing with the pitch and time interaction. For the original music, we fitted the TRF with two binary-coded regressors—note onset and downbeat (DBo). The TRF weights, as shown in Figure 3*a*, revealed that the waveform for note onset exhibited typical auditory evoked potential features, including P1 and P2 peaks, which appeared at ∼80 and 185 ms poststimulus, respectively. The TRF weights for downbeat regressor also demonstrated a peak at ∼185 ms and a dip at ∼650 ms, across most of the scalp electrodes, as shown in Figure 3*b* (permutation test with cluster).

We conducted the same analysis for chimeric music, albeit with four binary-coded regressors in fitting the TRF: note onset, downbeat-time (DBct), downbeat-pitch (DBcp), and the interaction (DBcpt). Please see Methods and Materials for a detailed description of these regressors. The TRF weights for note onset from chimeric music shared the same morphology as that of original music (Fig. 3*c*) and tested not significantly different (all *p* > 0.05, permutation test with cluster). Similarly, the TRF weights derived from DBct mirrored those of the DBo of original music (Fig. 3*d*), with no significant difference (all *p* > 0.05, permutation test with cluster).

### Downbeat encoding is also influenced by the pitch structure

Downbeats are defined by the temporal structure of the music. However in chimeric music, we can further investigate whether pitch structure influences this distinct downbeat neural response, via the downbeat-pitch (DBcp) and the pitch × time interaction regressors (DBcpt), specifically.

The derived TRF for DBcp regressor revealed a broad peak ∼260 ms on channel Fz. However, this observation did not reach statistical significance when compared with the null response, at Fz or other electrodes (Fig. 3*e*; all *p* > 0.05, permutation test with cluster). The TRF weights of the interaction term (Dcpt) demonstrated a peak at ∼180 ms (Fig. 3*f*), where there was an enhancement of P2 amplitude. This enhancement is in addition to the effect of timing downbeats. This result, combined with the lack of a significant pitch-downbeat response, supports the hypothesis that the neural coding of beat is driven by timing but influenced by the pitch structure (and pitch alone is not enough to indicate a downbeat). The cluster of significant channels and timepoints of the derived TRF can be found in supplemental materials (Figs. S2–S7).

To interpret these results, it is important to understand that the model is designed in an incremental fashion. For original stimuli, there are two regressors: note onset (for all notes of any type) and downbeats (DBo). As can be seen in Figure 1*c*, where there is an impulse in the DBo regressor, there is also one in the onset regressor. In this way, the generic note onset response (Fig. 3*a*) is already regressed out, and the DBo response (Fig. 3*b*) reflects the difference in the response to downbeats and all note onsets. We thus can recognize downbeat-specific processing by the existence of a DBo response. A brain that responded to notes but did not recognize downbeats would still show an onset response but no DBo response. The model is the same but more complex for chimeric music, where downbeat types are decoupled. There is a base note onset response, timing, and pitch-downbeat responses (DBct and DBcp, which show the difference between those downbeats and note onsets) and additional pitch × time interaction (DBcpt) that shows the additional response beyond the sum of the first three responses. This last component would be zero if the brain processed time and pitch downbeats separately.

**Figure 4.**
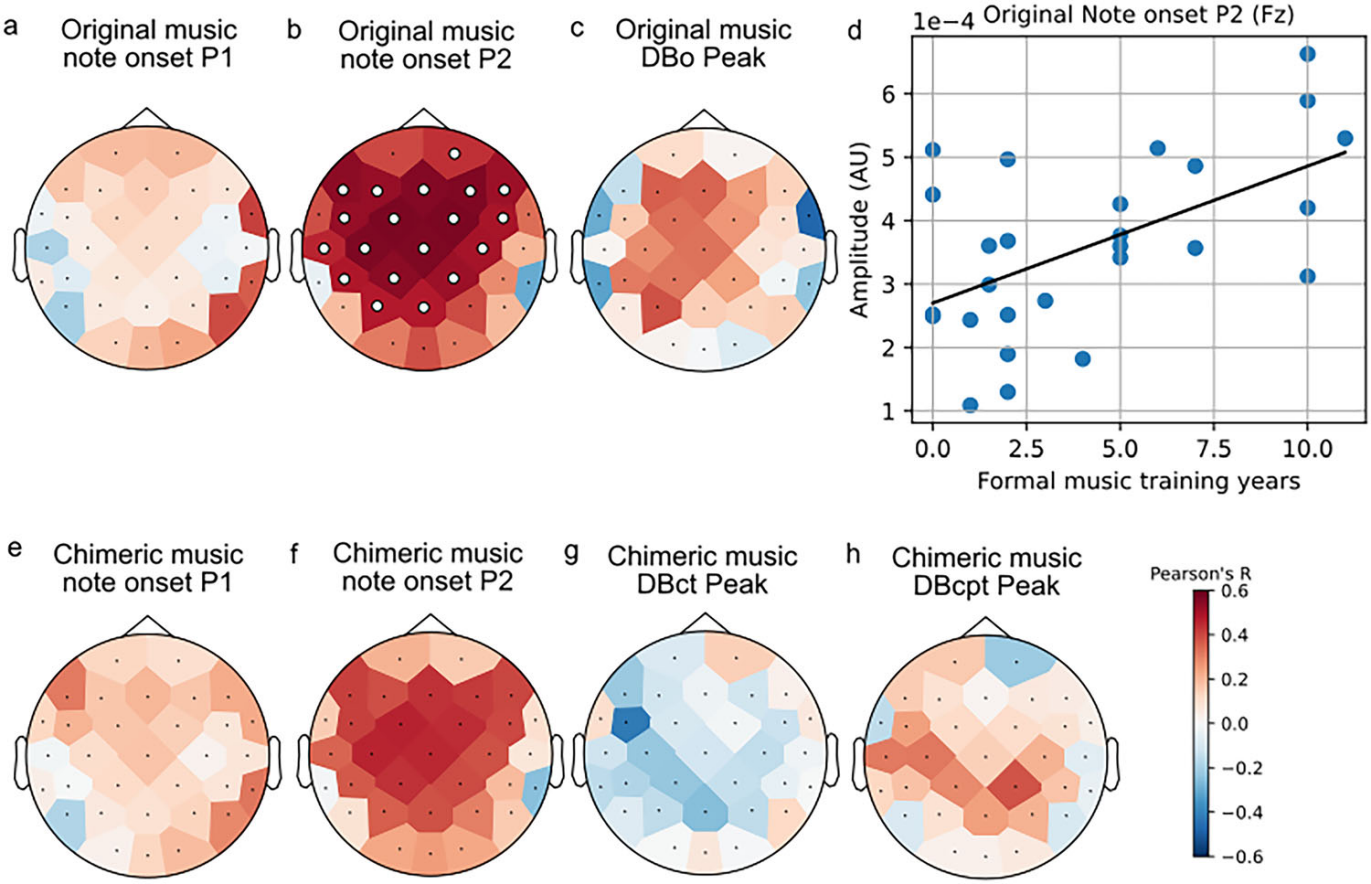
The correlation between the peak amplitude of the TRF weights for each regressor and the participants’ formal music training years. The larger circles indicate channels that have significant correlation. For the correlation on channel Fz (***d***), *r* = 0.55; *p* = 0.015 (FDR corrected across channels).

We also examined the impact of musicianship, measured by the formal music training years of the participants, which ranged from 0 to 11 years (4.19 ± 3.58, mean ± SD). To understand how musicianship influences the neural encoding of music, we used Pearson's correlation to assess the relationship between the years of musical training and the amplitudes of TRF weights at specific peak components—P1 and P2 for note onset regressors and P2 peak for downbeat-related regressors (DBo, DBct, and DBcpt) across all channels. Significant correlation was observed between the musical training and the maximum amplitude of the P2 component for the note onset regressor across the majority of channels (Fig. 4*b*,*d*). However, the maximum amplitude values from the TRF weights of other regressors did not exhibit a significant correlation with the musicianship (Fig. 4*a*,*c*,*e*–*h*).

### Subcortical responses are same for original and chimeric music

**Figure 5.**
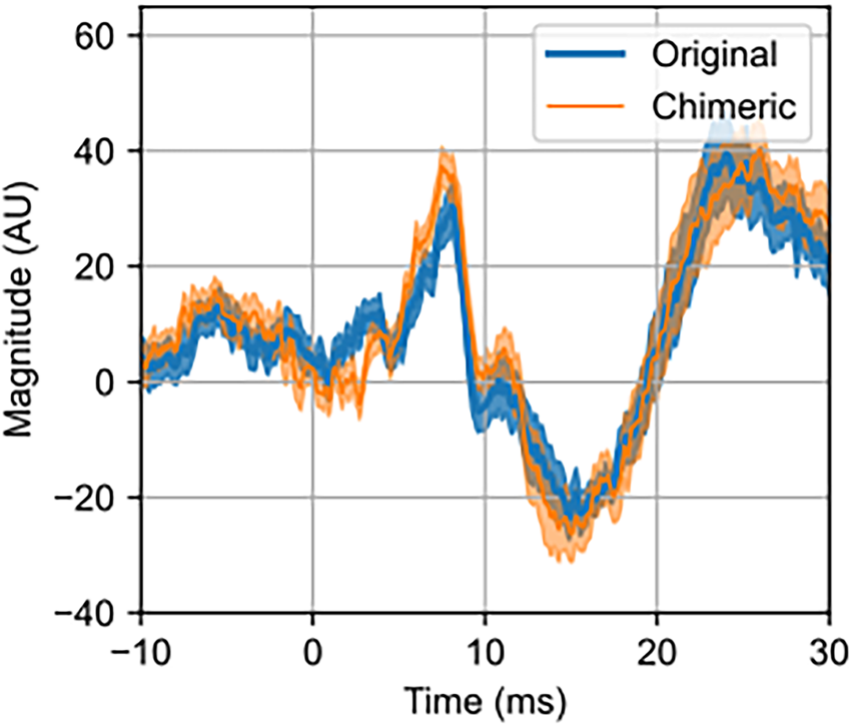
Grand-averaged ABR waveforms derived from original and chimeric music. Shaded area shows ±1 SEM.

We also investigated the ABRs as subcortical responses derived from the original and chimeric music, shown in Figure 5. The waveforms have the canonical ABR morphology, which is also consistent with previous study (Shan et al., 2024). The waveforms for original and chimeric music were highly correlated. No significant difference was found between the two stimulus categories (all *p* > 0.05, timepoint-wise Wilcoxon sign-rank test, FDR corrected).

## Discussion

By using chimeric music as a stimulus, we sought to investigate how pitch and time interact during music listening. We used TRF analyses that assess that neural tracking of continuous auditory stimuli as the main tool to study two aspects of music neural encoding: (1) melodic expectation encoding and its difference between original and chimeric music and (2) downbeat encoding and the pitch–time interaction influence on temporal structure perception.

For the melodic expectation encoding analysis, a notable advantage of using the chimeric music is that it preserved the context of each dimension, resulting in identical melodic expectation regressors to the original music while interrupting the linked role of pitch and time in building up the musical structure. Therefore, the TRF analysis and the comparison were well controlled.

For melodic expectation we found that, consistent with previous research (Di Liberto et al., 2020a; Kern et al., 2022; Bianco et al., 2024), a model that includes melodic expectation features has better predictive accuracy than the baseline acoustic-only model. This result also held true for chimeric music, even though the normal associations of pitch and temporal structure were violated (Fig. 2*b*). An interesting finding was that prediction accuracy was better overall for the chimeric music. We can only guess why this was the case, but it may be related to listening more intently to atypical melodies. A previous study found that atonal melodies with the same rhythm as typical Western ones showed stronger EEG tracking, which the authors attributed to less expected pitch sequences (Keitel et al., 2025).

The differences in prediction accuracy between the baseline acoustical model (A) and the model with melodic expectation features (AM) allowed us to determine the melodic expectation enhancement effect (*Δr*). This effect was greater for the original music than for chimeric music. It was also greater for the full model (AM-A) than for either the pitch-only or time-only models (AMp-A and AMt-A, respectively). These differences demonstrate that chimeric music's structural violations resulting from decoupling pitch and time indeed influenced melodic expectation encoding (Fig. 2*d*). The expectation enhancement effect was robust across training contexts: whether the model was trained only on the presented material (STM-only in IDyOM), on the full corpus excluding the presented material (LTM-only), or on the full corpus (“both”), we consistently observed a significant expectation enhancement effect (all analyses presented here are based on the “both” model). This indicates that the observed differences between original and chimeric music cannot be explained solely by the match between the training corpus and participants’ prior musical experience. Instead, these results suggest that structural disruptions in chimeric music themselves reduce the encoding of melodic expectations, above and beyond corpus-driven enculturation.

The neural tracking (*r*) of both baseline model (A) and melodic expectation model (AM) was significantly correlated with the length of musical training, aligned with previous studies where musicians have stronger encoding of music (Di Liberto et al., 2020a,b). However, unlike Di Liberto et al. (2020a), no correlation was found between musical training and the expectation enhancement effect (Δ*r*). This difference may arise from the fact that in that study there were discrete groups of musicians and nonmusicians, whereas we did not recruit in this way (that difference was not the focus of our study) and included the musical training information in our models for exploration. As a result, the variability in musical training is more diffusely distributed and may be diluted or have covaried with other individual difference factors (e.g., familiarity, preference), reducing the likelihood of detecting a robust group difference effect. Interestingly, there was no significant difference in TRF waveforms between original and chimeric music within the AM model (refer to Fig. S1), suggesting that while the effectiveness of expectation mechanisms may vary with the musical structures, the underlying neural encoding processes may remain consistent. A second explanation could be that the differences were small and distributed across the scalp, which would allow them to be seen in EEG prediction but not in the response waveforms at single electrodes.

In addition, an interesting observation we found in the mixed-effect linear models was that the preference of the song has an impact on the tracking of music in general (Table 1) and the expectation enhancement effect (Δ*r*) (Table 2), especially for chimeric music. Specifically, even though the more preferred chimeric music elicited reduced tracking of the acoustic features, they exhibited substantially stronger tracking of predictive expectations, producing a larger divergence between the AM and A models.

In our analysis on the downbeat encoding with TRF, the note onset regressor had a TRF similar to the canonical morphology of the auditory evoked potential (Picton et al., 1974; Lalor et al., 2009), characterized by P1 and P2 components. This onset-evoked potential was the same for original and chimeric music. Further exploration of downbeat encoding with TRF revealed that downbeat notes elicit neural responses distinct from nondownbeats. This distinction was evident in both original music with the DBo regressor (which reflects the coupled downbeat-pitch and time) and chimeric music with DBct regressor (which reflects downbeats defined by time). The neural downbeat response was enhanced ∼185 ms, reflecting a larger P2 peak for downbeats.

These two results together suggest the distinct neural responses elicited by downbeat notes in comparison with nondownbeat notes, highlighting the significance of downbeats in the cognitive processing of musical structures. This is also in line with the previous study where the accented stimuli showed an enhanced N1/P2 complex in perception than unaccented within a percussive rhythmic pattern (Abecasis et al., 2009; Schaefer et al., 2011). It should be noted, however, that all notes in our stimuli, including downbeats, were of equal level. In other words, downbeats were defined entirely by their musical context.

One novel aspect of this study is the delineation of pitch structure's impact on downbeat encoding. While the pitch component alone (Dcp) did not significantly affect the neural response to downbeats, the interaction between downbeat-pitch and downbeat-time term (Dcpt) showed a change in neural activity. This suggests that the cognitive processing of downbeats in music is multidimensional, where the interaction of the pitch and time structure of music both play a role.

The impact of musicianship on neural encoding of music was demonstrated by a significant correlation between years of formal musical training and the P2 amplitude for note onsets. The P2 component is often associated with auditory attention (Picton and Hillyard, 1974) and sensitive to neuroplasticity (Tremblay et al., 2001; Atienza et al., 2002; Shahin et al., 2003), indicating that more musically trained individuals may exhibit enhanced processing of novel musical stimuli (Shahin et al., 2003; Di Liberto et al., 2020b). However, unlike other studies that have reported differing rhythmic processing in musicians (Abecasis et al., 2009; Zhao et al., 2017), our results suggest that while musicianship appears to influence the neural encoding of basic auditory features such as note onsets, a uniform effect on the encoding of more complex musical structures such as downbeats may be harder to measure. We also suspect that while the note onset response from the downbeat analysis is a different calculation than the reconstruction accuracy calculated earlier in the paper, their respective correlations with musical training duration may reflect a single underlying relationship.

Another aspect of our results was the high correlation between the ABR waveforms generated by original and chimeric music, which aligns with the canonical ABR morphology, suggesting the subcortical level processing of auditory information was likely to be the same, regardless of the high level complexity of the musical stimuli (Shan et al., 2024).

A natural question that we do not address in this study is what brain regions and networks underlie the effects we observed here. Our 32-channel EEG setup offered limited spatial resolution, and the scalp topographies we observed for essentially all responses were a broad frontocentral activation that is seen widely in auditory studies. Prior work has suggested that spectral and temporal sensitivity are anatomically dissociated between cortical hemispheres (Schönwiesner and Zatorre, 2008). Hemispheric differences can be difficult to identify in auditory EEG due to auditory dipole orientations, and we did not find any asymmetry in our data (Fig. 3). Additionally, the basal ganglia and cerebellum have been shown to be involved in rhythm processing (Nozaradan et al., 2017; Kameda et al., 2023). Our measurements lacked the resolution to identify any responses generated in these deeper regions. Now that we have identified these interaction effects, a study using source-localized magnetoencephalography could allow these more detailed questions regarding their neural substrates to be answered. Future work leveraging the superior spatial resolution and sensitivity to subcortical activity provided by fMRI or intracranial EEG would also be valuable in this regard.

Using chimeric music to decouple the two fundamental dimensions of melody—pitch and time—we confirmed that the structure of each is processed by listeners and also found that their interaction is reflected in the listeners’ neural responses. This interaction plays a crucial role in developing the musical structure and pattern (Tillmann, 2012; Lerdahl, 2019) and thus impacts how music is perceived and processed by the human brain.

## Data Availability

The EEG recording data are available in EEG-BIDS format at OpenNeuro.org (https://openneuro.org/datasets/ds006735). The Python code for this study is available on GitHub (https://github.com/maddoxlab/Chimeric_music).

## References

[B1] Abecasis D, Brochard R, Del Río D, Dufour A, Ortiz T (2009) Brain lateralization of metrical accenting in musicians. Ann N Y Acad Sci 1169:74–78. 10.1111/j.1749-6632.2009.04766.x 19673756

[B2] Ablin P, Cardoso J-F, Gramfort A (2018) Faster independent component analysis by preconditioning with Hessian approximations. IEEE Trans Signal Process 66:4040–4049. 10.1109/TSP.2018.2844203

[B3] Atienza M, Cantero JL, Dominguez-Marin E (2002) The time course of neural changes underlying auditory perceptual learning. Learn Mem 9:138–150. 10.1101/lm.46502 12075002 PMC182592

[B4] Bialas O, Dou J, Lalor EC (2023) mTRFpy: a Python package for temporal response function analysis. J Open Source Softw 8:5657. 10.21105/joss.05657

[B5] Bianco R, Zuk NJ, Bigand F, Quarta E, Grasso S, Arnese F, Ravignani A, Battaglia-Mayer A, Novembre G (2024) Neural encoding of musical expectations in a non-human primate. Curr Biol 34:444–450.e5. 10.1016/j.cub.2023.12.019 38176416

[B6] Bjørndalen OM, Doursenaud R (2023) Mido - MIDI objects for Python. In (Version 1.3.2). Available at: https://mido.readthedocs.io/

[B7] Bouwer FL, Van Zuijen TL, Honing H (2014) Beat processing is pre-attentive for metrically simple rhythms with clear accents: an ERP study. PLoS One 9:e97467. 10.1371/journal.pone.0097467 24870123 PMC4037171

[B8] Crosse MJ, Di Liberto GM, Bednar A, Lalor EC (2016) The multivariate temporal response function (mTRF) toolbox: a MATLAB toolbox for relating neural signals to continuous stimuli. Front Hum Neurosci 10:604. 10.3389/fnhum.2016.00604 27965557 PMC5127806

[B9] Di Liberto GM, O'Sullivan JA, Lalor EC (2015) Low-frequency cortical entrainment to speech reflects phoneme-level processing. Curr Biol 25:2457–2465. 10.1016/j.cub.2015.08.030 26412129

[B10] Di Liberto GM, Pelofi C, Bianco R, Patel P, Mehta AD, Herrero JL, de Cheveigné A, Shamma S, Mesgarani N (2020a) Cortical encoding of melodic expectations in human temporal cortex. Elife 9:e51784. 10.7554/eLife.51784 32122465 PMC7053998

[B11] Di Liberto GM, Pelofi C, Shamma S, de Cheveigné A (2020b) Musical expertise enhances the cortical tracking of the acoustic envelope during naturalistic music listening. Acoust Sci Technol 41:361–364. 10.1250/ast.41.361

[B12] Ding N, Chatterjee M, Simon JZ (2014) Robust cortical entrainment to the speech envelope relies on the spectro-temporal fine structure. Neuroimage 88:41–46. 10.1016/j.neuroimage.2013.10.054 24188816 PMC4222995

[B13] Ding N, Simon JZ (2012) Neural coding of continuous speech in auditory cortex during monaural and dichotic listening. J Neurophysiol 107:78–89. 10.1152/jn.00297.2011 21975452 PMC3570829

[B14] Fitzroy AB, Sanders LD (2015) Musical meter modulates the allocation of attention across time. J Cogn Neurosci 27:2339–2351. 10.1162/jocn_a_00862 26284995 PMC6016835

[B15] Geiser E, Sandmann P, Jäncke L, Meyer M (2010) Refinement of metre perception–training increases hierarchical metre processing. Eur J Neurosci 32:1979–1985. 10.1111/j.1460-9568.2010.07462.x 21050278

[B16] Grahn JA (2012) Neural mechanisms of rhythm perception: current findings and future perspectives. Top Cogn Sci 4:585–606. 10.1111/j.1756-8765.2012.01213.x 22811317

[B17] Gramfort A, et al. (2013) MEG and EEG data analysis with MNE-Python. Front Neurosci 7:267. 10.3389/fnins.2013.00267 24431986 PMC3872725

[B18] Herbst SK, Obleser J (2019) Implicit temporal predictability enhances pitch discrimination sensitivity and biases the phase of delta oscillations in auditory cortex. Neuroimage 203:116198. 10.1016/j.neuroimage.2019.116198 31539590

[B19] Huron D (2008) Sweet anticipation: music and the psychology of expectation. Cambridge, Massachusetts: MIT Press.

[B20] Jehan T (2005) Downbeat prediction by listening and learning. *IEEE Workshop on Applications of Signal Processing to Audio and Acoustics, 2005*.

[B21] Jones MR, Boltz M, Kidd G (1982) Controlled attending as a function of melodic and temporal context. Percept Psychophys 32:211–218. 10.3758/BF03206225 7177759

[B22] Jones MR, Moynihan H, MacKenzie N, Puente J (2002) Temporal aspects of stimulus-driven attending in dynamic arrays. Psychol Sci 13:313–319. 10.1111/1467-9280.00458 12137133

[B23] Kameda M, Niikawa K, Uematsu A, Tanaka M (2023) Sensory and motor representations of internalized rhythms in the cerebellum and basal ganglia. Proc Natl Acad Sci U S A 120:e2221641120. 10.1073/pnas.2221641120 37276394 PMC10268275

[B24] Keitel A, Pelofi C, Guan X, Watson E, Wight L, Allen S, Mencke I, Keitel C, Rimmele J (2025) Cortical and behavioral tracking of rhythm in music: effects of pitch predictability, enjoyment, and expertise. Ann N Y Acad Sci 1546:120–135. 10.1111/nyas.15315 40101105 PMC11998481

[B25] Kern P, Heilbron M, de Lange FP, Spaak E (2022) Cortical activity during naturalistic music listening reflects short-range predictions based on long-term experience. Elife 11:e80935. 10.7554/eLife.80935 36562532 PMC9836393

[B26] Krumhansl CL (2000) Rhythm and pitch in music cognition. Psychol Bull 126:159. 10.1037/0033-2909.126.1.159 10668354

[B27] Lalor EC, Power AJ, Reilly RB, Foxe JJ (2009) Resolving precise temporal processing properties of the auditory system using continuous stimuli. J Neurophysiol 102:349–359. 10.1152/jn.90896.2008 19439675

[B28] Larson E, McCloy D, Maddox R, Pospisil D (2014) expyfun: Python experimental paradigm functions. In (Version 2.0.0). Available at: https://github.com/LABSN/expyfun/tree/2.0.0

[B29] Larson E, et al. (2023) MNE-Python. In (Version v1.6.0) Zenodo. 10.5281/zenodo.10161630

[B30] Lelo-de-Larrea-Mancera ES, Rodríguez-Agudelo Y, Solís-Vivanco R (2017) Musical rhythm and pitch: a differential effect on auditory dynamics as revealed by the N1/MMN/P3a complex. Neuropsychologia 100:44–50. 10.1016/j.neuropsychologia.2017.04.001 28389366

[B31] Lerdahl F (2019) Composition and cognition: reflections on contemporary music and the musical mind. Oakland, California: University of California Press.

[B32] Lerdahl F, Jackendoff RS (1996) A generative theory of tonal music, reissue, with a new preface. Cambridge, Massachusetts: MIT Press.

[B33] Luck SJ (2014) An introduction to the event-related potential technique. Cambridge, Massachusetts: MIT Press.

[B34] Marion G, Di Liberto GM, Shamma SA (2021) The music of silence: part I: responses to musical imagery encode melodic expectations and acoustics. J Neurosci 41:7435–7448. 10.1523/JNEUROSCI.0183-21.2021 34341155 PMC8412990

[B35] Moon IJ, Kang S, Boichenko N, Hong SH, Lee KM (2020) Meter enhances the subcortical processing of speech sounds at a strong beat. Sci Rep 10:15973. 10.1038/s41598-020-72714-z 32994430 PMC7525485

[B36] Nozaradan S, Schwartze M, Obermeier C, Kotz SA (2017) Specific contributions of basal ganglia and cerebellum to the neural tracking of rhythm. Cortex 95:156–168. 10.1016/j.cortex.2017.08.015 28910668

[B37] Pearce M, Wiggins G (2012) Auditory expectation: the information dynamics of music perception and cognition. Top Cogn Sci 4:625–652. 10.1111/j.1756-8765.2012.01214.x 22847872

[B38] Pearce MT (2005) The construction and evaluation of statistical models of melodic structure in music perception and composition. London, UK: City University London.

[B39] Picton TW, Hillyard SA, Krausz HI, Galambos R (1974) Human auditory evoked potentials. I: evaluation of components. Electroencephalogr Clin Neurophysiol 36:179–190. 10.1016/0013-4694(74)90155-2 4129630

[B40] Picton TW, Hillyard SA (1974) Human auditory evoked potentials. II: effects of attention. Electroencephalogr Clin Neurophysiol 36:191–200. 10.1016/0013-4694(74)90156-4 4129631

[B41] Prince JB, Schmuckler MA, Thompson WF (2009) The effect of task and pitch structure on pitch-time interactions in music. Mem Cognit 37:368–381. 10.3758/MC.37.3.368 19246351

[B42] Prince JB (2011) The integration of stimulus dimensions in the perception of music. Q J Exp Psychol 64:2125–2152. 10.1080/17470218.2011.573080 21598201

[B43] Schaefer RS, Vlek RJ, Desain P (2011) Decomposing rhythm processing: electroencephalography of perceived and self-imposed rhythmic patterns. Psychol Res 75:95–106. 10.1007/s00426-010-0293-4 20574661 PMC3036830

[B44] Schaffrath H (1995) The Essen folksong collection. Database containing 6.

[B45] Schönwiesner M, Zatorre RJ (2008) Depth electrode recordings show double dissociation between pitch processing in lateral Heschl’s gyrus and sound onset processing in medial Heschl’s gyrus. Exp Brain Res 187:97–105. 10.1007/s00221-008-1286-z 18236034

[B46] Seabold S, Perktold J (2010) Statsmodels: econometric and statistical modeling with python. SciPy 7:92–96. 10.25080/Majora-92bf1922-011

[B47] Shahin A, Bosnyak DJ, Trainor LJ, Roberts LE (2003) Enhancement of neuroplastic P2 and N1c auditory evoked potentials in musicians. J Neurosci 23:5545–5552. 10.1523/JNEUROSCI.23-13-05545.2003 12843255 PMC6741225

[B48] Shan T, Cappelloni MS, Maddox RK (2024) Subcortical responses to music and speech are alike while cortical responses diverge. Sci Rep 14:789. 10.1038/s41598-023-50438-0 38191488 PMC10774448

[B49] Snyder J, Krumhansl CL (2001) Tapping to ragtime: cues to pulse finding. Music Percept 18:455–489. 10.1525/mp.2001.18.4.455

[B50] Tierney A, Kraus N (2013) Neural responses to sounds presented on and off the beat of ecologically valid music. Front Syst Neurosci 7:14. 10.3389/fnsys.2013.00014 23717268 PMC3650712

[B51] Tierney A, Kraus N (2015) Neural entrainment to the rhythmic structure of music. J Cogn Neurosci 27:400–408. 10.1162/jocn_a_00704 25170794

[B52] Tillmann B (2012) Music and language perception: expectations, structural integration, and cognitive sequencing. Top Cogn Sci 4:568–584. 10.1111/j.1756-8765.2012.01209.x 22760955

[B53] Tillmann B, Bharucha JJ (2002) Effect of harmonic relatedness on the detection of temporal asynchronies. Percept Psychophys 64:640–649. 10.3758/BF03194732 12132764

[B54] Tremblay K, Kraus N, McGee T, Ponton C, Otis B (2001) Central auditory plasticity: changes in the N1-P2 complex after speech-sound training. Ear Hear 22:79–90. 10.1097/00003446-200104000-00001 11324846

[B55] Zhang J, Che X, Yang Y (2019) Event-related brain potentials suggest a late interaction of pitch and time in music perception. Neuropsychologia 132:107118. 10.1016/j.neuropsychologia.2019.107118 31176722

[B56] Zhao TC, Lam HG, Sohi H, Kuhl PK (2017) Neural processing of musical meter in musicians and non-musicians. Neuropsychologia 106:289–297. 10.1016/j.neuropsychologia.2017.10.007 28987905

